# Temporal associations between leukocytes DNA methylation and blood lipids: a longitudinal study

**DOI:** 10.1186/s13148-022-01356-x

**Published:** 2022-10-23

**Authors:** Zhiyu Wu, Lu Chen, Xuanming Hong, Jiahui Si, Weihua Cao, Canqing Yu, Tao Huang, Dianjianyi Sun, Chunxiao Liao, Yuanjie Pang, Zengchang Pang, Liming Cong, Hua Wang, Xianping Wu, Yu Liu, Yu Guo, Zhengming Chen, Jun Lv, Wenjing Gao, Liming Li

**Affiliations:** 1 Department of Epidemiology and Biostatistics, School of Public Health, Peking University, Beijing, 100191 China; 2 National Institute of Health Data Science at Peking University, Peking University, Beijing, 100191 China; 3 Peking University Center for Public Health and Epidemic Preparedness and Response, Beijing, 100191 China; 4 Qingdao Center for Disease Control and Prevention, Qingdao, 266033 China; 5 Zhejiang Center for Disease Control and Prevention, Hangzhou, 310051 China; 6 Jiangsu Center for Disease Control and Prevention, Nanjing, 210008 China; 7 Sichuan Center for Disease Control and Prevention, Chengdu, 610041 China; 8 Heilongjiang Center for Disease Control and Prevention, Harbin, 150090 China; 9 Fuwai hospital Chinese Academy of Medical Sciences, Beijing, 100037 China; 10 Clinical Trial Service Unit and Epidemiological Studies Unit (CTSU), Nuffield Department of Population Health, University of Oxford, Oxford, OX3 7LF UK; 11 Key Laboratory of Molecular Cardiovascular Sciences (Peking University), Ministry of Education, Beijing, 100191 China

**Keywords:** DNA methylation, Blood lipids, EWAS, Cross-lagged analysis, Mediation analysis

## Abstract

**Background:**

The associations between blood lipids and DNA methylation have been investigated in epigenome-wide association studies mainly among European ancestry populations. Several studies have explored the direction of the association using cross-sectional data, while evidence of longitudinal data is still lacking.

**Results:**

We tested the associations between peripheral blood leukocytes DNA methylation and four lipid measures from Illumina 450 K or EPIC arrays in 1084 participants from the Chinese National Twin Registry and replicated the result in 988 participants from the China Kadoorie Biobank. A total of 23 associations of 19 CpG sites were identified, with 4 CpG sites located in or adjacent to 3 genes (*TMEM49*, *SNX5/SNORD17* and *CCDC7*) being novel. Among the validated associations, we conducted a cross-lagged analysis to explore the temporal sequence and found temporal associations of methylation levels of 2 CpG sites with triglyceride and 2 CpG sites with high-density lipoprotein-cholesterol (HDL-C) in all twins. In addition, methylation levels of cg11024682 located in *SREBF1* at baseline were temporally associated with triglyceride at follow-up in only monozygotic twins. We then performed a mediation analysis with the longitudinal data and the result showed that the association between body mass index and HDL-C was partially mediated by the methylation level of cg06500161 (*ABCG1*), with a mediation proportion of 10.1%.

**Conclusions:**

Our study indicated that the DNA methylation levels of *ABCG1*, *AKAP1* and *SREBF1* may be involved in lipid metabolism and provided evidence for elucidating the regulatory mechanism of lipid homeostasis.

**Supplementary Information:**

The online version contains supplementary material available at 10.1186/s13148-022-01356-x.

## Background

An abnormal blood lipid profile, also known as dyslipidemia, is a major risk factor for atherosclerotic cardiovascular diseases such as myocardial infarction and stroke [1, 2], which mainly manifests as elevated plasma triglyceride (TG), total cholesterol (TC), low-density lipoprotein-cholesterol (LDL-C) levels and decreased high-density lipoprotein-cholesterol (HDL-C) levels. The prevalence of dyslipidemia contributes to a great global burden of disease. For example, elevated LDL-C levels were one of the top 10 risk factors for all-cause mortality and disability-adjusted life-years worldwide in 2019 [3].

Blood lipid levels are a complex trait regulated by various genetic and environmental factors [4–9], with DNA methylation being a potential regulatory mechanism. DNA methylation is the "annotation system" of gene sequences and affects gene expression by dynamically modifying the methylation or demethylation state of CpG sites on DNA. The methylation status of the same individual and the same CpG site may change over time. DNA methylation modification is related to the physiological effects of environmental factors on the human body and is also involved in the pathological process of disease occurrence and progression [10]. Therefore, DNA methylation may play an important role in maintaining blood lipid homeostasis.

Several epigenome-wide association studies (EWAS) have been conducted in predominantly European populations to explore the association between DNA methylation and blood lipid levels and have identified CpG sites on genes such as *ABCG1*, *CPT1A*, and *SREBF1* [11–15]. However, most existing studies have mostly been based on cross-sectional data to analyze the association between DNA methylation and blood lipids, which makes it difficult to assess the causal relationship between them. Mendelian randomization (MR) is an effective method for causal inference with cross-sectional data [16]. Some studies have used MR to estimate the causal relationships between DNA methylation levels and blood lipid levels [17–19]. However, the application of MR is limited because of the sample size and strict assumptions. Due to the variability of DNA methylation, studies based on longitudinal data are the best approach for causal inference.

As one of the risk factors for dyslipidemia, body mass index (BMI) is also associated with DNA methylation, and several CpG sites are associated with both BMI and blood lipids [20]. Previous studies proved that BMI could influence the methylation level of certain CpG sites [21–23], but whether DNA methylation mediates the association between BMI and blood lipids remains unclear.

Thus, we performed an EWAS of blood lipids with a discovery stage and independent replication in participants from the Chinese National Twin Registry (CNTR) and China Kadoorie Biobank (CKB). We assessed the temporal association between replicated CpG sites and lipids using the cross-lagged panel model (CLPM) in 308 participants with follow-up data from CNTR. For the CpG sites influencing blood lipids, we further conducted a mediation analysis with the CLPM to examine the role of DNA methylation in the association between BMI and blood lipids.

## Results

### Demographic

After quality control, 1060 participants in CNTR and 948 participants in CKB were retained in the EWAS (see Additional file 1: Table S1 for further details). The characteristics of the EWAS participants were shown in Table 1. The mean age of the two populations was around 50 years old (49.90 ± 12.15 and 50.56 ± 7.48). Males or never smokers accounted for more than half of the participants. In the discovery stage, approximately half of the people had never consumed alcohol, while in the replication stage, approximately 60% of the participants were current drinkers. Participants in the replication stage were more likely to have lower BMI, higher TG levels, and lower TC, HDL-C, and LDL-C levels than those in the discovery stage.

**Table 1:** Characteristics of participants in the EWAS phase

	EWAS		Temporal association	
	Discovery stage	Replication stage	Baseline	Follow-up
*N*	1060	948	288	
Age, yrs	49.90 ± 12.15	50.56 ± 7.48	49.60 ± 10.05	54.26 ± 10.02
*Sex, n (%)*				
Female	337 (31.8)	416 (43.9)	110 (38.2)	
Male	723 (68.2)	532 (56.1)	178 (61.8)	
MZ, *n* (%)	748 (70.6)	–	174 (60.4)	
*Fasting status, n (%)*				
≥ 8 h	–	123 (13.0)	–	
< 8 h	–	825 (87.0)	–	
*Smoking status, n (%)*				
Never	575 (54.2)	514 (54.2)	175 (60.8)	162 (56.3)
Former	136 (12.8)	51 (5.4)	25 (8.7)	45 (15.6)
Current	349 (32.9)	383 (40.4)	88 (30.6)	81 (28.1)
*Alcohol consumption, n (%)*				
Never	539 (50.8)	365 (38.5)	134 (46.5)	125 (43.4)
Former	73 (6.9)	15 (1.6)	9 (3.1)	82 (28.5)
Current	448 (42.3)	568 (59.9)	145 (50.3)	81 (28.1)
BMI, kg/m^2^	24.83 ± 3.91	23.57 ± 3.49	24.35 ± 3.56	24.46 ± 3.55
TG, mmol/L	1.85 ± 2.00	1.94 ± 1.18	1.86 ± 1.60	1.81 ± 1.34
TC, mmol/L	4.88 ± 1.04	4.58 ± 1.01	4.71 ± 0.98	4.79 ± 0.89
HDL-C, mmol/L	1.36 ± 0.38	1.21 ± 0.30	1.37 ± 0.32	1.29 ± 0.36
LDL-C, mmol/L	2.50 ± 0.77	2.28 ± 0.73	2.13 ± 0.61	2.72 ± 0.81

Continuous variables are expressed as mean ± SD and categorical variables are expressed as *n* (%)

*EWAS*, epigenome-wide association study; *MZ*, Monozygotic twins; *BMI*, body mass index; *TG*, triglyceride; *TC*, total cholesterol; *HDL-C*, high-density lipoprotein-cholesterol; *LDL-C*, low-density lipoprotein-cholesterol

### EWAS

For Model 1, the genomic inflation factors (*λ*) ranged between 1.009 and 1.049. We identified 26 CpG-lipid associations in the discovery stage, and methylation levels of 17, 3, 5, and 1 CpG sites were associated with TG, TC, HDL-C, and LDL-C, respectively (Additional file 1: Figure S1). A total of 23 associations of 19 CpG sites were replicated in the replication stage, and 15, 3, 5, and 0 CpG sites remained significant for the four lipid measures, respectively (Table 2). The direction of the effect was consistent in the two stages. cg06500151 (*ABCG1*), cg11024682 (*SREBF1*), cg19693031 (*TXNIP*) and cg27243685 (*ABCG1*) were associated with two lipid measures. In addition, we identified 4 novel CpG sites located in 2 genes (cg12054453 and cg18942579 in *TMEM49* and cg17507897 in *SNX5/SNORD17*) and 1 intergenic region (cg05176551 adjacent to *CCDC7*).

**Table 2:** Associations between DNA methylation and lipid measures (Model 1)

CpG	Discovery stage			Replication stage			Position	Gene
	*β*	SE	*P*_adj_	*β*	SE	*P*_adj_		
*TG*								
cg06500161	0.0130	0.0013	2.41E−14	0.0088	0.0013	1.51E−10	21:43,656,587	*ABCG1*
cg00574958	− 0.0074	0.0008	3.63E−12	− 0.0084	0.0010	1.20E−14	11:68,607,622	*CPT1A*
cg11024682	0.0085	0.0011	1.77E−09	0.0059	0.0011	9.33E−08	17:17,730,094	*SREBF1*
cg19693031	− 0.0211	0.0027	2.41E−09	− 0.0110	0.0022	1.15E−06	1:145,441,552	*TXNIP*
cg17058475	− 0.0084	0.0011	4.34E−08	− 0.0100	0.0012	5.70E−16	11:68,607,737	*CPT1A*
cg07504977	0.0102	0.0014	6.70E−07	0.0066	0.0016	3.47E−05	10:102,131,012	*OLMALINC*
cg27243685	0.0090	0.0013	3.88E−06	0.0084	0.0014	4.42E−09	21:43,642,366	*ABCG1*
cg01176028	0.0055	0.0009	5.82E−04	0.0047	0.0010	1.88E−06	21:43,653,234	*ABCG1*
cg05778424	0.0078	0.0014	7.36E−04	0.0057	0.0012	7.46E−06	17:55,169,508	*AKAP1*
cg08857797	0.0087	0.0016	3.16E−03	0.0073	0.0016	5.06E−06	17:40,927,699	*VPS25*
cg26403843	0.0108	0.0020	3.96E−03	0.0062	0.0024	1.11E−02	5:158,634,085	*RNF145*
cg06690548	− 0.0089	0.0017	1.44E–02	− 0.0037	0.0013	6.10E−03	4:139,162,808	*SLC7A11*
cg09737197	− 0.0059	0.0012	2.13E−02	− 0.0068	0.0012	7.32E−08	11:68,607,675	*CPT1A*
cg20544516	0.0054	0.0011	2.40E−02	0.0066	0.0011	7.27E−09	17:17,717,183	*MIR33B; SREBF1*
**cg05176551**	0.0079	0.0016	3.24E−02	0.0064	0.0014	5.83E−06	10:32,701,586	*CCDC7*
*TC*								
**cg12054453**	0.0395	0.0060	5.95E−05	0.0154	0.0059	1.22E−02	17:57,915,717	*TMEM49*
cg19693031	− 0.0416	0.0078	2.76E−02	− 0.0286	0.0062	1.62E−05	1:145,441,552	*TXNIP*
**cg18942579**	0.0278	0.0053	4.23E−02	0.0138	0.0055	1.22E−02	17:57,915,773	*TMEM49*
*HDL-C*								
cg06500161	− 0.0218	0.0031	4.23E−06	− 0.0204	0.0032	9.44E−10	21:43,656,587	*ABCG1*
cg17901584	0.0243	0.0043	3.01E−03	0.0305	0.0048	9.44E−10	1:55,353,706	*DHCR24*
cg11024682	− 0.0138	0.0024	3.01E−03	− 0.0122	0.0027	6.44E−06	17:17,730,094	*SREBF1*
cg27243685	− 0.0167	0.0030	6.39E−03	− 0.0166	0.0034	2.05E−06	21:43,642,366	*ABCG1*
**cg17507897**	− 0.0179	0.0033	1.07E−02	− 0.0093	0.0034	5.96E−03	20:17,943,694	*SNX5; SNORD17*

The novel CpG sites are in bold font. *P*-values are adjusted for FDR

*β*, regression coefficient; *SE*, standard error; *TG*, triglyceride; *TC*, total cholesterol; *HDL-C*, high-density lipoprotein-cholesterol

When we further adjusted for BMI in Model 2, only 1/3 (8/23) of the associations were robust. The number of significant CpG sites was reduced by half for TG (7/15) and TC (1/3), and no CpG site was associated with HDL-C (Additional file 1: Table S2). The effect estimates were attenuated except for cg05176551. Two of the four novel CpG sites (cg05176551 for TG and cg12054453 for TC) remained significant.

### Enrichment analysis

The results of enrichment analyses based on the EWAS from model 1 were shown in Additional file 1: Table S3–S6. GO enrichment analysis for TG revealed lipid and cellular ketone metabolic pathways. Insulin and glucose metabolism, peptide synthesis, and regulatory processes were also enriched in the analysis, which appeared to be related to TG homeostasis. Sterol signaling pathway, as well as alcohol signaling pathway, were enriched in the GO enrichment analysis for HDL. KEGG and Reactome analysis suggested enrichment for pathways associated with liver disease, lipid metabolism, and antioxidants such as PPARalpha and HMOX1, although the pathways did not survive after multiple comparisons (FDR > 0.05).

### Cross-lagged analysis

Our cross-lagged analysis showed that two CpG sites had significant temporal associations with TG and two had significant temporal associations with HDL-C in all twins (Table 3). The paths of TG at baseline to the methylation levels of cg27243685 and cg05778424 at follow-up (*ρ*_1_) were significant, suggesting that the TG level had an effect on these CpG sites. We observed the same direction of association between HDL-C and the methylation level of cg11024682. The reverse path (*ρ*_2_) was significant for cg06500161 and HDL-C, suggesting that this CpG site could influence HDL-C levels. We did not detect any temporal associations between CpG sites and TC.

**Table 3:** Cross-lagged association between lipid measures and DNA methylation

CpG	Gene	Lipid_baseline_ → Methylation_follow-up_			Methylation_baseline_ → Lipid_follow-up_			Model fit	
		*β*	SE	*P*_adj_	*β*	SE	*P*_adj_	SRMR	CFI
*TG*									
cg27243685	*ABCG1*	0.3884	0.1426	**0.0483**	0.0264	0.0220	0.6682	< 0.001	1
cg05778424	*AKAP1*	0.5423	0.1660	**0.0163**	0.0147	0.0209	0.9468	< 0.001	1
*HDL-C*									
cg06500161	*ABCG1*	− 0.5781	0.5812	0.3999	− 0.0320	0.0088	**0.0014**	< 0.001	1
cg11024682	*SREBF1*	− 1.4046	0.4655	**0.0127**	− 0.0193	0.0121	0.1837	< 0.001	1

Adjusted *P*-values less than 0.05 are in bold font

*β*, regression coefficient; *SE*, standard error; *TG*, triglyceride; *HDL-C*, high-density lipoprotein-cholesterol; *SRMR*, standardized root mean squared residual; *CFI*, comparative fit index

We then performed a stratification analysis and revealed some new temporal associations. In monozygotic (MZ) twins, the methylation level of cg11024682 at baseline showed a significant association with TG at follow-up (*ρ*_2_). In dizygotic (DZ) twins, cg17507897 also showed an association between HDL-C at baseline and methylation level at follow-up (*ρ*_1_). The associations between cg05778424, cg06500161, and cg11024682 with TG or HDL-C could still be observed in only DZ twins (Additional file 1: Table S8).

### Mediation analysis

**Fig. 1:**
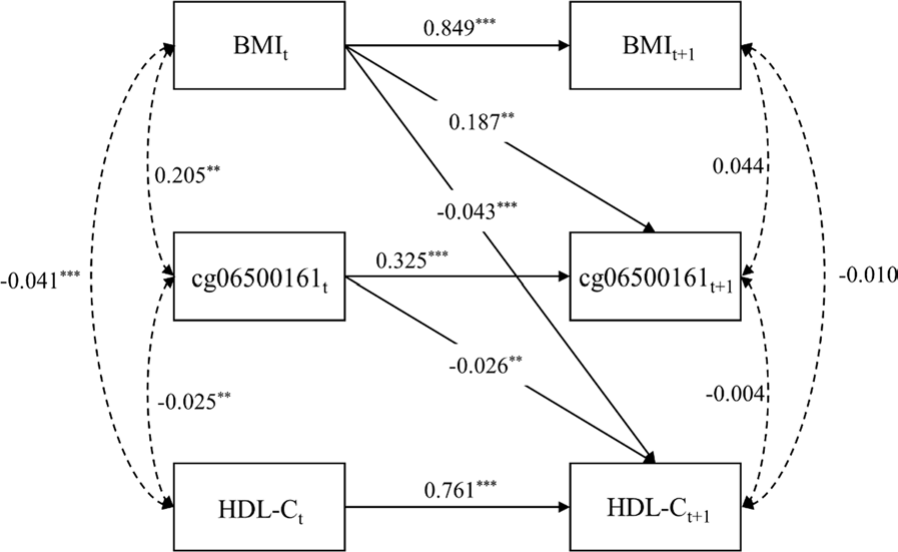
Cross-lagged panel model about the mediation effect of cg06500161 between BMI and HDL-C. The subscript *t* indicates traits at baseline, and the subscript *t* + *1* indicates traits at follow-up. Model fit: SRMR = 0.004, CFI = 1.000. ^*^P < 0.05; ^**^P < 0.01; ^***^P < 0.001

According to the results of the cross-lagged analysis, cg06500161 and cg11024682, which had effects on HDL-C or TG, were selected as potential mediators in the mediation analysis. We first estimated the temporal association between BMI and lipid or CpG sites using the CLPM. In all twins, BMI at baseline was associated with TG, HDL-C, and the methylation level of the two CpG sites at follow-up (Table 4). We then performed a mediation analysis with the methylation level of CpG sites as the mediator, BMI as exposure, and lipid measures as the outcome, and only the mediating effect of cg06500161 was observed. The results are shown in Fig. 1. BMI was associated with HDL-C with a significant direct effect, indirect effect, and total effect (all *P* < 0.05). The methylation level of cg06500161 explained 10.1% (*P* < 0.05) of the association between BMI and HDL-C.

**Table 4:** Cross-lagged association between BMI and lipid measures or between BMI and DNA methylation

Lipid measures or CpG	Gene	BMI_baseline_ → Trait_follow-up_			Trait_baseline_ → BMI_follow-up_			Model fit	
		*β*	SE	*P*	*β*	SE	*P*	SRMR	CFI
*BMI and lipid measures*									
TG	–	0.0675	0.0199	**0.0007**	0.0698	0.1133	0.5376	< 0.001	1
HDL	–	− 0.0467	0.0113	** < 0.0001**	− 0.0009	0.2667	0.9972	< 0.001	1
*BMI and CpG sites*									
cg06500161	*ABCG1*	0.1853	0.0558	**0.0018**	− 0.0019	0.0387	0.9600	< 0.001	1
cg11024682	*SREBF1*	0.1062	0.0512	**0.0380**	0.0237	0.0428	0.9600	< 0.001	1

Adjusted *P*-values less than 0.05 are in bold font

*β*, regression coefficient; *SE*, standard error; *TG*, triglyceride; *HDL-C*, high-density lipoprotein-cholesterol; *SRMR*, standardized root mean squared residual; *CFI*, comparative fit index

## Discussion

In the current study, we reported the associations between lipid measures and DNA methylation and inferred the potential causal direction of the associations. To our knowledge, this is the first EWAS of blood lipids reported in the Chinese population. We identified 23 associations of 19 CpG sites, and 4 CpG sites located in or adjacent to 3 genes were novel. With the cross-lagged panel model, we found potential causal relationships of 2 CpG sites with TG and 2 CpG sites with HDL-C among all the significant associations and a relationship of cg11024682 with TG in only MZ twins. In addition, we conducted a mediation analysis and found that BMI has an effect on HDL-C, and the methylation level of cg06500161 partially mediates this effect.

In our EWAS, we confirmed that the widely validated CpG sites in European populations, such as cg06500161 in *ABCG1*, cg00574958 in *CPT1A*, and cg11024682 in *SREBF1*, were also associated with lipid measures robustly in East Asian populations and the direction of the associations were consistent. This complemented the findings of Jhun et al. [24] that the associations between blood lipids and DNA methylation have consistency across ethnic groups, including Europeans, African Americans, and Hispanics. Another study compared differences in DNA methylation patterns of multiple traits related to non-communicable diseases between black South Africans and Europeans and found that 95% confidence intervals for effect estimates overlapped for more than 85% of the shared lipid-related CpG sites [25]. However, we still identified 3 novel genes with TG (*CCDC7*), TC (*TMEM49*), and HDL-C (*SNX5/SNORD17*) with moderate sample size, and the latter two remained robust after adjustment for BMI. The differences in the methylation patterns may be associated with the genetic background of different ethnic groups [25], but the exact mechanisms need to be further explored.

Two CpG sites located in *TMEM49* were related to TC. *TMEM49*, also known as *VMP1*, encodes an endoplasmic reticulum (ER) transmembrane protein that regulates the formation of autophagosomes, lipid droplets, lipoproteins, and other ER-derived structures [26, 27]. *TMEM49* has been reported to affect the activity of lipoprotein-associated phospholipase A (2) (Lp-PLA2) [28], an inflammatory enzyme that is a risk factor for coronary heart disease and in which lipids may play a role [29, 30]. Lp-PLA2 was bound to serum cholesterol, and in vitro experiments showed that elevated LDL-C levels result in upregulation of Lp-PLA2 [31]. All these findings indicated a potential role for *TMEM49* in TC homeostasis. In addition, the methylation level of *TMEM49* was also associated with waist circumference [32], survival of cancer overall [33], and chemotherapy in breast cancer patients [34].

SNX5, short for sorting nexin 5, is a key regulator of endosomal trafficking [35], and *SNX5* has been identified as an LDL-C-associated gene [36]. Elevated or decreased expression of *SNX5* was found in multiple cancers [37, 38], and renal *SNX5* was reported to positively regulate insulin-degrading enzyme expression and function [39]. Noncoding small nucleolar RNA SNORD17 is also related to carcinogenesis, including hepatocellular carcinoma [40], cervical cancer [41], and colon adenocarcinoma [42]. The association of *SNX5/SNORD17* with HDL-C needs to be further investigated.

Previous studies have reported an association between BMI and some lipid-related CpG sites [20, 43, 44], which was validated to some extent in our Model 2 results. The magnitude of the association between lipid measures and methylation was attenuated when adjusting for BMI, indicating that BMI may partially influence the association. None of the HDL-C-related CpG sites remained significant. Given that these CpG sites were also BMI-related, we hypothesized that BMI might affect HDL-C through the CpG sites. This hypothesis was partially confirmed by the subsequent mediation analysis.

Based on the results of the cross-lagged analysis, we found that the methylation levels of *ABCG1* could influence HDL-C levels and be influenced by TG levels. Previous studies have explored the causal relationship between *ABCG1* methylation and TG and HDL-C using MR and gene expression analysis methods and obtained opposite directions [17, 45, 46]. Min et al. and Dekkers et al. [17] both found that the methylation level of *ABCG1* was influenced by TG or HDL-C using MR analysis, while Pfeiffer et al. [46] suggested that the methylation level of *ABCG1* could regulate TG and HDL-C levels through gene expression. Since our study used longitudinal data while the three studies above were based on cross-sectional data, we believe that our results are more credible. The ATP-binding cassette transporter (ABCG1) is the critical mediator of reverse cholesterol transport (RCT) and mediates cellular cholesterol efflux to HDL particles [47–50]. The methylation level of cg06500161 (*ABCG1*) was correlated with the *ABCG1* transcript level, which in turn had an impact on HDL-C [46].

Our results also showed that the methylation level of *AKAP1* might be influenced by TG. A-kinase anchoring protein 121 (AKAP1) binds protein kinase A and anchors it to the mitochondrial outer membrane to maintain mitochondrial function [51]. Animal experiments have demonstrated that *AKAP1* is involved in the regulation of endothelial cell behavior [52], oxidative stress, and apoptosis [53] and plays a role in multiple metabolism-related diseases. For instance, researchers observed that energy expenditure and thermogenesis were significantly enhanced in brown adipose tissue of *AKAP1* knockout obese mice, which could attenuate diet-induced obesity and insulin resistance [54]. DNA methylation may be an intermediate link in the association of *AKAP1* with these diseases, and TG, as one of the important metabolic indicators, may have a similar regulatory mechanism.

In the stratification analysis, a noteworthy result was that cg11024682 located in *SREBF1* had a potential influence on TG levels only in MZ twins. The direction of the association between TG and *SREBF1* in our results was not completely consistent with a previous study, which found that the methylation level of *SREBF1* was influenced by TG using MR analysis [17]. We hypothesize that the inconsistent results are due to genetic and early-life environmental factors. MR analysis used genetic variants as instrumental variables and could not exclude confounding from genetic factors. Since MZ twins share almost 100% genetic background and early-life environment, our result, which was found only in MZ twins, suggests that genetic and early-life environmental factors may influence the effects of the CpG site on TG. *SREBF1* encodes sterol regulatory element-binding protein 1, which can activate and synthesize cholesterol and fatty acids [55]. MicroRNA (miR)-33b, located in the intron region of *SREBF1*, can also act as an important regulator of lipid metabolism [56, 57]. Antagonism of miR-33 inhibited the expression of genes involved in fatty acid synthesis, such as *SREBF1*, and thus reduced plasma very low-density lipoprotein triglyceride levels [58].

Our mediation analysis supported that cg06500161 (*ABCG1*) partially mediated the effect of BMI on HDL-C. Previous studies have reported that the methylation level of cg06500161 could be influenced by BMI [21–23] and mediate the effect of BMI on the expression of *ABCG1* [59]. Johansson and his colleagues found that *ABCG1* and *CETP* were the most upregulated genes that were differentially expressed in obese patients during weight loss and weight maintenance after weight loss, and the prevailing HDL concentration was correlated with the expression of *ABCG1* [60]. Similar results were observed in morbidly obese women [61]. However, the above two studies only analyzed *ABCG1* expression levels in adipose tissue rather than blood samples. Therefore, the mediating role of *ABCG1* and its methylation level between BMI and HDL-C remains to be further explored.

The strength of our study is that we explored the temporal association between CpG sites and blood lipids using longitudinal data and corresponding analysis, circumventing the limitations of cross-sectional data in causal inference. In addition, the analysis in twins, especially MZ twins, provides a natural matched design to present causal inference while controlling for genetic and early family environmental factors [62].

There are also some limitations of our study. In the EWAS phase, to maximize the sample size, only probes common to both the 450 K and EPIC methylation arrays were included in our study, while other probes present in only one methylation array were excluded. Some potential lipid-associated CpG sites may not have been identified. In addition, the CKB cohort did not require participants to fast prior to blood sample collection, which may have affected the validation results. Therefore, we adjusted the fasting status in our analysis and used SmartSVA to control for potential confounding, avoiding the interference of fasting time on the results to some extent. In the cross-lagged analysis phase, the power of the stratification analysis was limited due to the moderate sample size. However, we still found some evidence that the association between TG and *SREBF1* may be influenced by genetic and early-life environmental factors.

## Conclusions

We identified 4 novel CpG sites related to lipid measures in the Chinese population. Based on the longitudinal twin data, we found the temporal sequence of DNA methylation levels of *ABCG1*, *AKAP1,* and *SREBF1* with TG and HDL-C. In addition, we observed a potential mediation role of cg06500161 (*ABCG1*) in the temporal association between BMI and HDL-C. Our study provides evidence to elucidate the underlying biological mechanisms of DNA methylation in lipid metabolism, and future studies should continue to explore the biological role of DNA methylation in lipid metabolism and downstream effects on disease.

## Method

### Study population

The discovery stage of the EWAS phase and cross-lagged analysis phase was based on CNTR. Established in 2001, CNTR is a population-based twin registry, and the details of CNTR have been reported previously [63]. The current study included participants who participated in the follow-up surveys in 2013–2014 and 2017–2018. A total of 1392 blood specimens were collected for methylation measurements from 1084 participants, including 308 participants with repeated measurements. The replication stage of EWAS was based on CKB, a prospective cohort of more than 0.5 million adults in 10 geographically defined regions across China since 2004–2008 [64, 65]. DNA methylation data were available for 988 participants selected for a case‒control study nested within CKB [66]. Information from the two cohorts was collected via standardized questionnaires, physical examinations, and blood biochemical examinations.

### Data measurements

Lipid measures, including TG, TC, HDL-C, and LDL-C, were measured in blood samples. Participants were asked to fast for at least 8 h in CNTR, while fasting was not mandatory in CKB. The fasting time for each participant was recorded and adjusted in the subsequent analysis in CKB. Other covariates, such as age, sex, smoking status, and alcohol consumption, were collected using the interview-administered questionnaire. Smoking status and alcohol consumption were divided into three categories: never, former and current. Height and weight were collected in the physical examination to calculate BMI, which was defined as weight in kilograms divided by the square of height in meters. Medication history was also recorded, and participants taking lipid-lowering medicine were excluded. Outliers that were three standard deviations from the mean of lipid measures in each cohort were removed. Blood samples with missing biochemical parameters or moderate to severe lipemia were also excluded. All lipid measures were natural log-transformed to approximate a normal distribution.

The zygosity of twins was determined based on the correlation of 59 SNPs in both the 450 K and EPIC methylation arrays. It was shown that the possible cutoff point was between 0.84 and 0.90 [67], which was set to 0.90 in the current study. Twin pairs with a correlation coefficient higher than 0.90 were considered MZ twins; otherwise, they were considered DZ twins.

### DNA methylation and quality control

Genomic DNA was extracted from peripheral blood leukocytes and bisulfite-converted using the EZ DNA methylation kit (Zymo Research, Orange, CA, USA). Epigenome-wide DNA methylation levels were measured using the Infinium HumanMethylation450 BeadChip assay (Illumina, San Diego, CA, USA) or Infinium HumanMethylationEPIC BeadChip assay (Illumina, San Diego, CA, USA), and only the overlapping CpG sites of the two assays were included in the subsequent procedure.

DNA methylation measurement and preprocessing were conducted independently in the two cohorts. In CNTR, we applied the R package *minfi* [68] to process and combine the raw methylation data of two assays and obtained the *β*-value of each CpG site to report the methylation level. For quality control, we removed the probes if they (1) had detection p values > 0.05 in more than 1% of samples or had bead counts < 3 in more than 5% of samples; (2) were non-CpG or multi-hit probes; (3) were related to SNPs with MAF > 0.05 in the 1000 Genomes Project for the East Asian population; and (4) were located in sexual chromosomes. We excluded samples if they (1) were sex mismatched and (2) had a detection *P*-value greater than 0.01. The details of quality control of the raw methylation data in CKB can be found in a previous study [66]. The stratified quantile normalization method [69] in the *minfi* package was used for preprocessing and normalization. The cell proportions for each cell type were estimated using Houseman’s method [70] and adjusted using the champ.refbase function in the R package ChAMP. To correct batch effects, we performed an optimized surrogate variable analysis (SVA) with the package SmartSVA [71] in the EWAS phase. SmartSVA provided a fast and robust method to remove potential confounding factors for epigenetic or other genomic studies and was developed based on the linear model. In the cross-lagged analysis phase, batch effects were corrected using the Combat method instead.

Finally, 378,654 CpG sites were retained in the discovery stage of EWAS. We annotated CpG sites to genes with the manifest file provided on the Illumina website. The CpG sites located in the intergenic region would be annotated to the nearest gene using the R package matchGenes or genome browser (https://genome.ucsc.edu/).

### Statistical analysis

#### EWAS

We implemented a two-stage EWAS with participants in CNTR and CKB. For Model 1, linear mixed regression models were fitted with *β*-value at a CpG site as the dependent variable and each lipid measure (TG, TC, HDL-C, and LDL-C) as the independent variable using the R package nlme, adjusting for age, sex, smoking status, alcohol consumption, and all surrogate variables generated above as fixed effects. Due to the correlation within twin pairs, the unique ID of each twin pair was added to the model as a random effect in the discovery stage. The fasting status was further adjusted as a fixed effect (< 8 or ≥ 8 h) in the replication stage. BMI was further adjusted in Model 2.

### Enrichment analysis

For all CpG sites associated with lipid measures, we then performed Gene Ontology (GO) term analyses, Kyoto Encyclopedia of Genes and Genomes (KEGG) enrichment analyses, and Reactome pathway enrichment analysis with the R package "methylGSA" [72].

### Cross-lagged analysis

We conducted a cross-lagged analysis based on participants with repeated measurements in CNTR, of whom the average follow-up duration was 4.67 ± 0.22 years. A residual analysis was performed first. Lipid measures were adjusted for age, sex, smoking status, and alcohol consumption as fixed effects at the corresponding time point and twin number as a random effect using the linear mixed regression model. The *β*-value of significant CpG sites was further adjusted with the blood cell proportions and batch effects. Residuals from the regression model were used in the subsequent cross-lagged analysis, and residuals of CpG sites were normalized using Z-transform. The CLPM simultaneously estimated the autoregressive and cross-lagged regressive effects of lipid measure and methylation data at two-time points, including (1) autoregression of lipid measure at follow-up on lipid measure at baseline, (2) autoregression of *β* value at follow-up on *β* value at baseline, (3) cross-lagged regression of *β*-value at follow-up on lipid measure at baseline (*ρ*_1_), and (4) cross-lagged regression of lipid measure at follow-up on *β*-value at baseline (*ρ*_2_). The significance and magnitude of *ρ*_1_ and *ρ*_2_ reflected the temporal associations of the two variables. We fitted a structural equation model to estimate all the parameters and statistics above using the R package *lavaan* and set the cluster argument to adjust for the correlation of twins. We also reported model fit indexes to evaluate model fit, including standardized root mean squared residual (SRMR) and comparative fit index (CFI), and models with SRMR < 0.08 and CFI > 0.95 were considered good fits [73]. We repeated the steps above in only MZ or DZ, and only twin pairs with complete data at two-time points were included.

### Mediation analysis

Since BMI was associated with DNA methylation and could affect blood lipids, we performed mediation analysis to assess whether CpG sites were the mediator of the effect of BMI on lipids. Only CpG sites that showed a potential effect on lipid measures in the cross-lagged analysis were included.

The temporal associations between BMI and lipid measures and between BMI and CpG sites were assessed with the CLPM first to deduce the potential mediator. The relationships among exposure *X*, mediator *M* and outcome *Y* at baseline (*b*) and follow-up (*f*) were as follows:

1 $$X_{f} = \beta_{X} X_{b} + \varepsilon_{Xf}$$

2 $$M_{f} = \beta_{M} M_{b} + aX_{b} + \varepsilon_{Mf}$$

3 $$Y_{f} = \beta_{Y} Y_{b} + bM_{b} + c^{\prime } X_{b} + \varepsilon_{Yf}$$

where *β* is the autoregressive coefficient, *ε* is the residual item, ab is the direct effect, and $c^{\prime }$ is the indirect effect. The model fitting and parameter estimation method was similar to that of the cross-lagged analysis.

The false discovery rate (FDR) was used for multiple comparisons, and the threshold of significance was considered to be less than 0.05. Statistical analysis was performed using R version 4.0.2 or 4.0.3.

## Supplementary Information

**Additional file 1**: **Table S1**. Details of sample size and quality control. **Figure S1**. Manhattan plots of the epigenome-wide association study (Model 1). Manhattan plots for (a) triglyceride (TG), (b) total cholesterol (TC), (c) high-density lipoprotein-cholesterol (HDL-C), and (d) low-density lipoprotein-cholesterol (LDL-C) in Model 1. The red horizontal dashed lines represent the FDR-adjusted threshold of significance. **Table S2**. Associations between DNA methylation and lipid measures (Model 2). **Table S3**. Enriched GO terms based on the results of TG EWAS. **Table S4**. Enriched GO terms based on the Model 1 results of HDL EWAS. **Table S5**. KEGG enriched pathways based on the results of EWAS. **Table S6**. Reactome enriched pathways based on the results of EWAS. **Table S8**. Cross-lagged association between lipid measures and DNA methylation stratified by zygosity.

## Data Availability

The datasets used and/or analysed during the current study are available from the corresponding author on reasonable request.

## Abbreviations

ABCG1: ATP-binding cassette transporter
AKAP1: A-kinase anchoring protein 121
BMI: Body mass index
CFI: Comparative fit index
CKB: China Kadoorie Biobank
CLPM: Cross-lagged panel model
CNTR: Chinese national twin registry
DZ: Dizygotic twin
ER: Endoplasmic reticulum
EWAS: Epigenome-wide association study
FDR: False discovery rate
GO: Gene ontology
HDL-C: High-density lipoprotein-cholesterol
KEGG: Kyoto encyclopedia of genes and genomes
LDL-C: Low-density lipoprotein-cholesterol
Lp-PLA2: Lipoprotein-associated phospholipase A (2)
MR: Mendelian randomization
MZ: Monozygotic twin
RCT: Reverse cholesterol transport
SNORD17: Small nucleolar RNA, C/D box 17
SNX5: Sorting nexin 5
SREBF1: Sterol regulatory element-binding protein 1
SRMR: Standardized root mean squared residual
SVA: Surrogate variable analysis
TC: Total cholesterol
TG: Triglyceride
TMEM49: Transmembrane protein 49
