# Supplementary material for: Temporal associations between leukocytes DNA methylation and blood lipids: a longitudinal study
Source: Clin Epigenetics. 2022 Oct 23;14:132. doi: 10.1186/s13148-022-01356-x (PMC9588246; doi:10.1186/s13148-022-01356-x)
Supplement: Supplementary file 1 — Additional file 1: Table S1. Details of sample size and quality control. Figure S1. Manhattan plots of the epigenome-wide association study (Model 1). Manhattan plots for (a) triglyceride (TG), (b) total cholesterol (TC), (c) high-density lipoprotein-cholesterol (HDL-C), and (d) low-density lipoprotein-cholesterol (LDL-C) in Model 1. The red horizontal dashed lines represent the FDR-adjusted threshold of significance. Table S2. Associations between DNA methylation and lipid measures (Model 2). Table S3. Enriched GO terms based on the results of TG EWAS. Table S4. Enriched GO terms based on the Model 1 results of HDL EWAS. Table S5. KEGG enriched pathways based on the results of EWAS. Table S6. Reactome enriched pathways based on the results of EWAS. Table S8. Cross-lagged association between lipid measures and DNA methylation stratified by zygosity. [file 13148_2022_1356_MOESM1_ESM.docx]

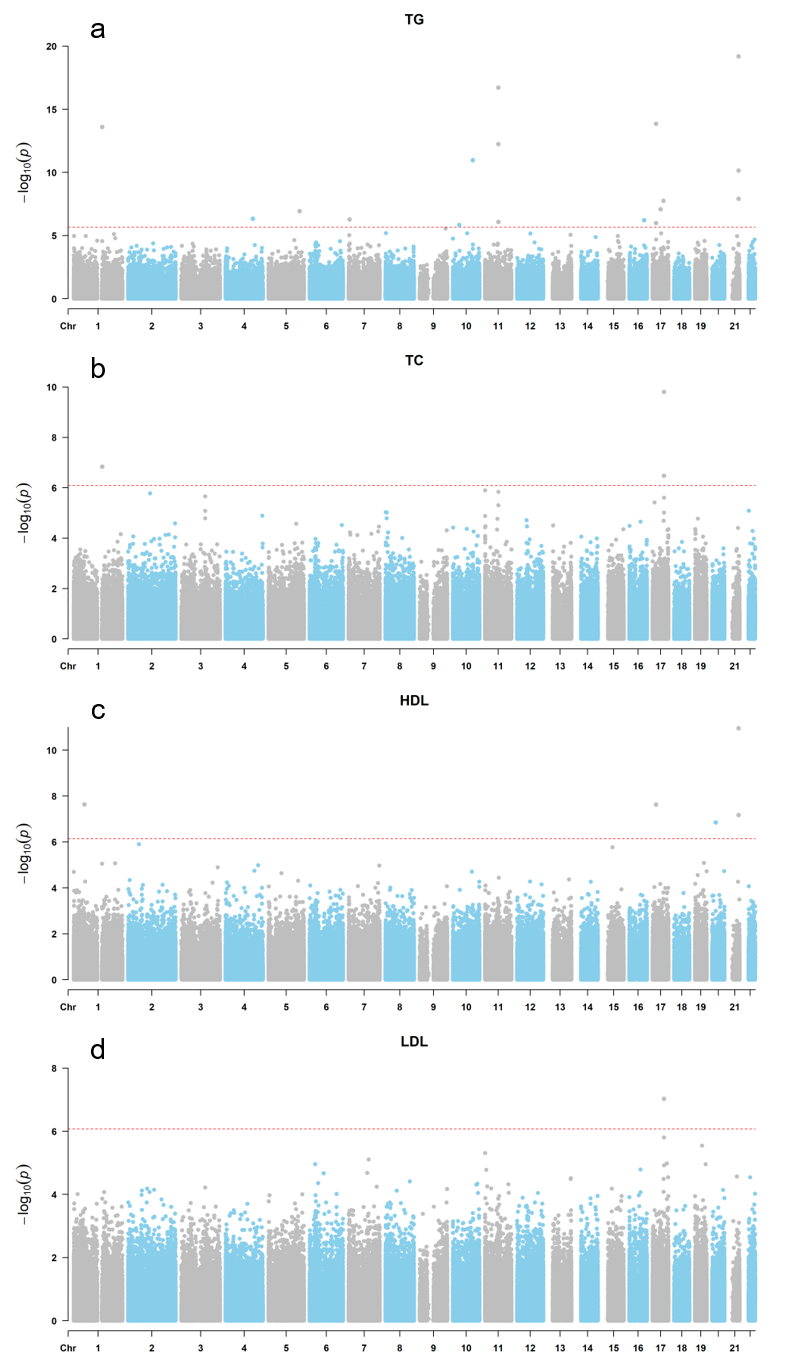


**Supplementary Figure 1**

**Manhattan plots of the epigenome-wide association study (Model 1).** Manhattan plots for (a) triglyceride (TG), (b) total cholesterol (TC), (c) high-density lipoprotein-cholesterol (HDL-C), and (d) low-density lipoprotein-cholesterol (LDL-C) in Model 1. The red horizontal dashed lines represent the FDR-adjusted threshold of significance.

**Supplementary Table 1 Details of sample size and quality control**

|  | **Discovery stage**  **of EWAS** | **Replication stage**  **of EWAS** | **Temporal association** |
| --- | --- | --- | --- |
| **Total population** | 1084 | 988 | 308 |
| Taking lipid-lowering medicine | 10 | 0 | 9 |
| Blood samples with moderate to severe lipemia | -^*^ | 32 | - |
| QC exclusions |  |  |  |
| Gender-mismatched | 10 | 2 | 0 |
| With detection p-value greater than 0.01 | 4 | 2 | 1 |
| Measured in a distinct study batch | - | 2 | - |
| Outliers detected by weighted gene co-methylation network analysis | - | 2 | - |
| Unpaired Twins | - | - | 10 |
| **Samples in the analysis** | 1060 | 948 | 288 |

^*^ Not applicable

EWAS, epigenome-wide association study; QC, quality control.

**Supplementary Table 2 Associations between DNA methylation and lipid measures (Model 2)**

| **CpG** | **Discovery Stage** | | | **Replication Stage** | | | **Position** | **Gene** | **Gene Group** | **Enhancer** |
| --- | --- | --- | --- | --- | --- | --- | --- | --- | --- | --- |
|  | **β** | **SE** | ***P*_adj_** | **β** | **SE** | ***P*_adj_** |  |  |  |  |
| **TG** |  |  |  |  |  |  |  |  |  |  |
| cg00574958 | -0.0063 | 0.0009 | 7.02E-07 | -0.0063 | 0.0011 | 3.20E-08 | 11:68607622 | *CPT1A* | 5'UTR |  |
| cg19693031 | -0.0205 | 0.0029 | 7.02E-07 | -0.0082 | 0.0023 | 6.02E-04 | 1:145441552 | *TXNIP* | 3'UTR |  |
| cg06500161 | 0.0101 | 0.0014 | 7.02E-07 | 0.0063 | 0.0014 | 1.21E-05 | 21:43656587 | *ABCG1* | Body | TRUE |
| cg17058475 | -0.0074 | 0.0012 | 1.95E-04 | -0.0081 | 0.0012 | 3.08E-10 | 11:68607737 | *CPT1A* | 5'UTR |  |
| cg11024682 | 0.0068 | 0.0011 | 2.95E-04 | 0.0038 | 0.0011 | 1.01E-03 | 17:17730094 | *SREBF1* | Body |  |
| cg07504977 | 0.0088 | 0.0015 | 1.49E-03 | 0.0042 | 0.0016 | 1.40E-02 | 10:102131012 | *OLMALINC* | Intergenic region | TRUE |
| **cg05176551** | 0.0087 | 0.0017 | 3.67E-02 | 0.0053 | 0.0014 | 6.02E-04 | 10:32701586 | *CCDC7* | Intergenic region | TRUE |
| **TC** |  |  |  |  |  |  |  |  |  |  |
| cg12054453 | 0.0386 | 0.0060 | 1.45E-04 | 0.0140 | 0.0060 | 2.07E-02 | 17:57915717 | *TMEM49* | Body |  |

The novel CpG site is in bold font. *P*-value are adjusted for FDR.

β, regression coefficient; SE, standard error; TG, triglyceride; TC, total cholesterol.

**Supplementary Table 3**

**Enriched GO terms based on the results of TG EWAS.**

| **ID** | **Description** | **Size** | ***P***  **Model 1** | ***P*_adj_**  **Model 1** | ***P***  **Model 2** | ***P*_adj_**  **Model 2** |
| --- | --- | --- | --- | --- | --- | --- |
| GO:0062012 | regulation of small molecule metabolic process | 313 | 9.59E-07 | 1.55E-03 | 2.62E-06 | 4.24E-03 |
| GO:0015833 | peptide transport | 257 | 2.40E-05 | 1.86E-02 | 1.55E-04 | 1.47E-02 |
| GO:0042886 | amide transport | 291 | 3.46E-05 | 1.86E-02 | 1.91E-04 | 1.48E-02 |
| GO:0010565 | regulation of cellular ketone metabolic process | 125 | 6.25E-05 | 2.52E-02 | 9.54E-04 | 4.05E-02 |
| GO:0062013 | positive regulation of small molecule metabolic process | 135 | 8.88E-05 | 2.87E-02 | 1.59E-05 | 1.10E-02 |
| GO:0045834 | positive regulation of lipid metabolic process | 144 | 1.29E-04 | 3.46E-02 | 2.81E-05 | 1.10E-02 |
| GO:0042180 | cellular ketone metabolic process | 202 | 1.82E-04 | 3.56E-02 | 1.63E-03 | 6.14E-02 |
| GO:0050796 | regulation of insulin secretion | 161 | 2.03E-04 | 3.56E-02 | 4.64E-05 | 1.10E-02 |
| GO:0051223 | regulation of protein transport | 497 | 2.28E-04 | 3.56E-02 | 7.04E-04 | 3.66E-02 |
| GO:1901617 | organic hydroxy compound biosynthetic process | 226 | 3.04E-04 | 3.56E-02 | 2.61E-03 | 8.44E-02 |
| GO:0090276 | regulation of peptide hormone secretion | 190 | 3.05E-04 | 3.56E-02 | 6.68E-05 | 1.10E-02 |
| GO:0030073 | insulin secretion | 190 | 3.20E-04 | 3.56E-02 | 7.16E-05 | 1.10E-02 |
| GO:0002791 | regulation of peptide secretion | 194 | 3.20E-04 | 3.56E-02 | 6.94E-05 | 1.10E-02 |
| GO:0090087 | regulation of peptide transport | 196 | 3.27E-04 | 3.56E-02 | 7.02E-05 | 1.10E-02 |
| GO:0009749 | response to glucose | 204 | 3.31E-04 | 3.56E-02 | 6.84E-05 | 1.10E-02 |
| GO:0009746 | response to hexose | 211 | 3.57E-04 | 3.57E-02 | 7.26E-05 | 1.10E-02 |
| GO:0034284 | response to monosaccharide | 217 | 3.76E-04 | 3.57E-02 | 7.50E-05 | 1.10E-02 |
| GO:0030072 | peptide hormone secretion | 229 | 5.11E-04 | 4.23E-02 | 1.09E-04 | 1.26E-02 |
| GO:0009743 | response to carbohydrate | 245 | 5.37E-04 | 4.23E-02 | 1.08E-04 | 1.26E-02 |
| GO:0046883 | regulation of hormone secretion | 241 | 5.39E-04 | 4.23E-02 | 1.07E-04 | 1.26E-02 |
| GO:0002790 | peptide secretion | 235 | 5.50E-04 | 4.23E-02 | 1.17E-04 | 1.26E-02 |

*P*-value are adjusted for FDR.

**Supplementary Table 4**

**Enriched GO terms based on the Model 1 results of HDL EWAS.**

| **ID** | **Description** | **Size** | ***P***  **Model 1** | ***P*_adj_**  **Model 1** |
| --- | --- | --- | --- | --- |
| GO:0008203 | cholesterol metabolic process | 132 | 4.29E-06 | 2.53E-03 |
| GO:0046165 | alcohol biosynthetic process | 134 | 4.65E-06 | 2.53E-03 |
| GO:1902652 | secondary alcohol metabolic process | 141 | 4.88E-06 | 2.53E-03 |
| GO:0016125 | sterol metabolic process | 146 | 6.28E-06 | 2.53E-03 |
| GO:0006694 | steroid biosynthetic process | 163 | 8.81E-06 | 2.84E-03 |
| GO:1901617 | organic hydroxy compound biosynthetic process | 226 | 2.34E-05 | 6.31E-03 |
| GO:0008202 | steroid metabolic process | 300 | 5.10E-05 | 1.18E-02 |
| GO:0006066 | alcohol metabolic process | 334 | 5.92E-05 | 1.19E-02 |

*P*-value are adjusted for FDR.

**Supplementary Table 5**

**KEGG enriched pathways based on the results of EWAS.**

| **ID** | **Description** | **Size** | ***P***  **Model 1** | ***P*_adj_**  **Model 1** | ***P***  **Model 2** | ***P*_adj_**  **Model 2** |
| --- | --- | --- | --- | --- | --- | --- |
| **TG** |  |  |  |  |  |  |
| path:hsa04931 | Insulin resistance | 105 | 2.44E-03 | 1.44E-01 | 9.83E-04 | 5.65E-02 |
| path:hsa04936 | Alcoholic liver disease | 127 | 2.69E-03 | 1.44E-01 | 8.31E-04 | 5.65E-02 |
| path:hsa04152 | AMPK signaling pathway | 118 | 3.77E-03 | 1.44E-01 | 1.74E-03 | 6.69E-02 |
| path:hsa04621 | NOD-like receptor signaling pathway | 158 | 8.33E-02 | 1.00E+00 | 4.21E-02 | 1.00E+00 |
| path:hsa04932 | Non-alcoholic fatty liver disease | 147 | 8.59E-02 | 1.00E+00 | 4.68E-02 | 1.00E+00 |
| **TC** |  |  |  |  |  |  |
| path:hsa04621 | NOD-like receptor signaling pathway | 158 | 1.82E-02 | 1.00E+00 | 1.00E+00 | 1.00E+00 |
| path:hsa04140 | Autophagy - animal | 133 | 2.23E-02 | 1.00E+00 | 1.15E-02 | 1.00E+00 |
| **HDL** |  |  |  |  |  |  |
| path:hsa04936 | Alcoholic liver disease | 127 | 3.39E-02 | 1.00E+00 | - | - |
| path:hsa04932 | Non-alcoholic fatty liver disease | 147 | 3.52E-02 | 1.00E+00 | - | - |
| path:hsa04931 | Insulin resistance | 105 | 3.60E-02 | 1.00E+00 | - | - |
| path:hsa04910 | Insulin signaling pathway | 131 | 4.39E-02 | 1.00E+00 | - | - |
| path:hsa04152 | AMPK signaling pathway | 118 | 4.55E-02 | 1.00E+00 | - | - |

*P*-value are adjusted for FDR.

**Supplementary Table 6**

**Reactome enriched pathways based on the results of EWAS.**

| **ID** | **Description** | **Size** | ***P***  **Model 1** | ***P*_adj_**  **Model 1** | ***P***  **Model 2** | ***P*_adj_**  **Model 2** |
| --- | --- | --- | --- | --- | --- | --- |
| **TG** |  |  |  |  |  |  |
| R-HSA-9006931 | Homo sapiens: Signaling by Nuclear Receptors | 224 | 4.61E-04 | 7.61E-02 | 9.50E-05 | 1.57E-02 |
| R-HSA-1989781 | Homo sapiens: PPARA activates gene expression | 113 | 2.53E-03 | 1.45E-01 | 8.88E-04 | 5.15E-02 |
| R-HSA-400206 | Homo sapiens: Regulation of lipid metabolism by PPARalpha | 115 | 2.64E-03 | 1.45E-01 | 9.36E-04 | 5.15E-02 |
| R-HSA-9707564 | Homo sapiens: Cytoprotection by HMOX1 | 114 | 6.80E-02 | 1.00E+00 | 3.80E-02 | 1.00E+00 |
| R-HSA-8957322 | Homo sapiens: Metabolism of steroids | 140 | 7.86E-02 | 1.00E+00 | 4.35E-02 | 1.00E+00 |
| R-HSA-9711123 | Homo sapiens: Cellular response to chemical stress | 144 | 8.39E-02 | 1.00E+00 | 4.66E-02 | 1.00E+00 |
| **TC** |  |  |  |  |  |  |
| R-HSA-9707564 | Homo sapiens: Cytoprotection by HMOX1 | 114 | 1.12E-02 | 1.00E+00 | 1.00E+00 | 1.00E+00 |
| R-HSA-9711123 | Homo sapiens: Cellular response to chemical stress | 144 | 1.40E-02 | 1.00E+00 | 1.00E+00 | 1.00E+00 |
| R-HSA-9658195 | Homo sapiens: Leishmania infection | 241 | 2.95E-02 | 1.00E+00 | 1.00E+00 | 1.00E+00 |
| **HDL** |  |  |  |  |  |  |
| R-HSA-8957322 | Homo sapiens: Metabolism of steroids | 140 | 5.41E-04 | 8.93E-02 | - | - |
| R-HSA-9006931 | Homo sapiens: Signaling by Nuclear Receptors | 224 | 2.13E-03 | 1.75E-01 | - | - |
| R-HSA-1989781 | Homo sapiens: PPARA activates gene expression | 113 | 3.18E-02 | 1.00E+00 | - | - |
| R-HSA-400206 | Homo sapiens: Regulation of lipid metabolism by PPARalpha | 115 | 3.27E-02 | 1.00E+00 | - | - |

*P*-value are adjusted for FDR.

**Supplementary Table 7**

**Characteristics of participants stratified by zygosity in the temporal association phase**

|  | **MZ** | **DZ** | ***P*** |
| --- | --- | --- | --- |
| N | 174 | 114 |  |
| Sex, n (%) |  |  |  |
| Female | 66 (37.9) | 44 (38.6) |  |
| Male | 108 (62.1) | 70 (61.4) | 0.668 |
| **Baseline** | | | |
| Age, yrs | 49.46±9.48 | 49.82±10.89 | 0.832 |
| Smoking status, n (%) | | | |
| Never | 105 (60.3) | 70 (61.4) |  |
| Former | 20 (11.5) | 5 (4.4) | 0.111 |
| Current | 49 (28.2) | 39 (34.2) | 0.566 |
| Alcohol consumption, n (%) | | | |
| Never | 83 (47.7) | 51 (44.7) |  |
| Former | 7 (4.0) | 2 (1.8) | 0.365 |
| Current | 84 (48.3) | 61 (53.5) | 0.564 |
| BMI, kg/m^2^ | 24.45±3.54 | 24.19±3.60 | 0.667 |
| TG, mmol/L | 1.97±1.87 | 1.69±1.04 | 0.255 |
| TC, mmol/L | 4.65±1.04 | 4.79±0.90 | 0.324 |
| HDL-C, mmol/L | 1.35±0.32 | 1.40±0.31 | 0.242 |
| LDL-C, mmol/L | 2.09±0.63 | 2.20±0.57 | 0.216 |
| **Follow-up** | | | |
| Age, yrs | 54.11±9.47 | 54.49±10.83 | 0.827 |
| Smoking status, n (%) | | | |
| Never | 97 (55.7) | 65 (57.0) |  |
| Former | 28 (16.1) | 17 (14.9) | 0.791 |
| Current | 49 (28.2) | 32 (28.1) | 0.937 |
| Alcohol consumption, n (%) | | | |
| Never | 78 (44.8) | 47 (41.2) |  |
| Former | 53 (30.5) | 29 (25.4) | 0.766 |
| Current | 43 (24.7) | 38 (33.3) | 0.268 |
| BMI, kg/m^2^ | 24.47±3.48 | 24.43±3.67 | 0.952 |
| TG, mmol/L | 1.83±1.38 | 1.79±1.29 | 0.808 |
| TC, mmol/L | 4.71±0.87 | 4.93±0.90 | 0.088 |
| HDL-C, mmol/L | 1.28±0.34 | 1.31±0.40 | 0.611 |
| LDL-C, mmol/L | 2.68±0.81 | 2.79±0.82 | 0.395 |

Continuous variables are expressed as mean ± SD and categorical variables are expressed as n (%). P-values are calculated with mixed effect model.

MZ, monozygotic twins; DZ, dizygotic twins; BMI, body mass index.

**Supplementary Table 8 Cross-lagged association between lipid measures and DNA methylation stratified by zygosity**

| CpG | Gene | Lipid_baseline_ → Methylation_follow-up_ | | | Methylation_baseline_ → Lipid_follow-up_ | | | Model fit | |
| --- | --- | --- | --- | --- | --- | --- | --- | --- | --- |
|  |  | β | SE | *P*_adj_ | β | SE | *P*_adj_ | SRMR | CFI |
| **MZ** |  |  |  |  |  |  |  |  |  |
| **TG** |  |  |  |  |  |  |  |  |  |
| cg11024682 | *SREBF1* | 0.2077 | 0.3107 | 0.9224 | 0.0816 | 0.0269 | **0.0362** | <0.001 | 1 |
| **DZ** |  |  |  |  |  |  |  |  |  |
| **TG** |  |  |  |  |  |  |  |  |  |
| cg05778424 | *AKAP1* | 0.6022 | 0.1573 | **0.0019** | -0.0049 | 0.0309 | 0.9740 | <0.001 | 1 |
| **HDL-C** |  |  |  |  |  |  |  |  |  |
| cg06500161 | *ABCG1* | -1.0689 | 0.6207 | 0.1417 | -0.0668 | 0.0177 | **0.0008** | <0.001 | 1 |
| cg11024682 | *SREBF1* | -1.5523 | 0.3482 | **<0.0001** | -0.0189 | 0.0215 | 0.6266 | <0.001 | 1 |
| cg17507897 | *SNX5; SNORD17* | -1.8686 | 0.5364 | **0.0012** | 0.0134 | 0.0205 | 0.6266 | <0.001 | 1 |

Adjusted *P*-values less than 0.05 are in bold font.

β, regression coefficient; SE, standard error; SRMR, standardized root mean squared residual; CFI, comparative fit index; MZ, monozygotic twins; DZ, dizygotic twins; TG, triglyceride; HDL-C, high-density lipoprotein-cholesterol.

**Supplementary Table 9 Cross-lagged association between BMI and lipid measures stratified by zygosity**

| Lipid measures | BMI_baseline_ → Lipid_follow-up_ | | | Lipid_baseline_ → BMI_follow-up_ | | | Model Fit | |
| --- | --- | --- | --- | --- | --- | --- | --- | --- |
|  | β | SE | *P* | β | SE | *P* | SRMR | CFI |
| **MZ** |  |  |  |  |  |  |  |  |
| TG | -0.0006 | 0.0267 | 0.9828 | -0.0204 | 0.2829 | 0.9424 | <0.001 | 1 |
| HDL | -0.0210 | 0.0109 | 0.0541 | -0.1449 | 0.5188 | 0.7801 | <0.001 | 1 |
| **DZ** |  |  |  |  |  |  |  |  |
| TG | 0.1486 | 0.0423 | **0.0004** | 0.0480 | 0.1218 | 0.6937 | <0.001 | 1 |
| HDL | -0.0664 | 0.0228 | **0.0036** | 0.3267 | 0.3036 | 0.2820 | <0.001 | 1 |

*P*-values less than 0.05 are in bold font.

β, regression coefficient; SE, standard error; SRMR, standardized root mean squared residual; CFI, comparative fit index; MZ, monozygotic twins; DZ, dizygotic twins; TG, triglyceride; HDL-C, high-density lipoprotein-cholesterol.

**Supplementary Table 10 Cross-lagged association between BMI and DNA methylation stratified by zygosity**

| CpG | Gene | BMI_baseline_ → Methylation_follow-up_ | | | Methylation_baseline_ → BMI_follow-up_ | | | Model Fit | |
| --- | --- | --- | --- | --- | --- | --- | --- | --- | --- |
|  |  | β | SE | *P*_adj_ | β | SE | *P*_adj_ | SRMR | CFI |
| **MZ** |  |  |  |  |  |  |  |  |  |
| cg06500161 | *ABCG1* | 0.0858 | 0.0908 | 0.3442 | 0.0027 | 0.0699 | 0.9692 | <0.001 | 1 |
| cg11024682 | *SREBF1* | -0.0906 | 0.0889 | 0.3442 | 0.0294 | 0.0760 | 0.9692 | <0.001 | 1 |
| **DZ** |  |  |  |  |  |  |  |  |  |
| cg06500161 | *ABCG1* | 0.1791 | 0.0777 | **0.0212** | -0.0109 | 0.0165 | 0.5603 | <0.001 | 1 |
| cg11024682 | *SREBF1* | 0.1759 | 0.0676 | **0.0185** | 0.0085 | 0.0146 | 0.5603 | <0.001 | 1 |

Adjusted *P*-values less than 0.05 are in bold font.

β, regression coefficient; SE, standard error; SRMR, standardized root mean squared residual; CFI, comparative fit index.
